# Evaluation of Defensins as Markers of Gut Microbiota Disturbances in Children with Obesity and Metabolic Dysfunction-Associated Steatotic Liver Disease (MASLD)

**DOI:** 10.3390/jcm14103505

**Published:** 2025-05-16

**Authors:** Aldona Wierzbicka-Rucińska, Ewa Konopka, Sebastian Więckowski, Wojciech Jańczyk, Anna Świąder-Leśniak, Jolanta Świderska, Joanna Trojanek, Zbigniew Kułaga, Piotr Socha, Joanna Bierła

**Affiliations:** 1 Department of Clinical Biochemistry, The Children’s Memorial Health Institute, Aleja Dzieci Polskich 20, 04-730 Warsaw, Poland; e.konopka@ipczd.pl; 2 Deparment of Gastroenterology, Hepatology and Nutrition Disorders, The Children’s Memorial Health Institute, Aleja Dzieci Polskich 20, 04-730 Warsaw, Poland; s.wieckowski@ipczd.pl (S.W.); w.janczyk@ipczd.pl (W.J.); p.socha@ipczd.pl (P.S.); 3 Laboratory of Anthropology, The Children’s Memorial Health Institute, Al. Dzieci Polskich 20, 04-730 Warsaw, Poland; a.swiader-lesniak@ipczd.pl; 4 Clinic of Endocrinology and Diabetology, The Children’s Memorial Health Institute, Aleja Dzieci Polskich 20, 04-730 Warsaw, Poland; j.swiderska@ipczd.pl; 5 Department of Microbiology and Clinical Immunology, The Children’s Memorial Health Institute, Al. Dzieci Polskich 20, 04-730 Warsaw, Poland; j.trojanek@ipczd.pl (J.T.); j.bierla@ipczd.pl (J.B.); 6 Department of Public Health, The Children’s Memorial Health Institute, Al. Dzieci Polskich 20, 04-730 Warsaw, Poland; z.kulaga@ipczd.pl

**Keywords:** defensins 5 and 6, intestinal fatty acid-binding protein (I-FABP), obesity, MASLD

## Abstract

Until recently, it was believed that bacterial translocation occurs as a result of leaky gut syndrome or sepsis. To confirm or exclude the process of bacterial translocation, biomarkers can be used. One such biomarker is defensins, which indicate immune activity, as defensins are cationic peptides with antibacterial properties produced by intestinal epithelial cells. Also, fatty acid-binding proteins (I-FABP and L-FABP) can serve as useful serological markers for intestinal epithelial damage, indicating impaired intestinal permeability or organ damage, as high concentrations of them are found in tissues and low concentrations in blood serum. In the context of obesity, the integrity of the intestinal barrier, which can be disrupted by dietary fat, leads to increased intestinal permeability. Since bacterial translocation and microbiota contribute to obesity and Metabolic Dysfunction-Associated Steatotic Liver Disease (MASLD) associated with metabolic dysfunction, intestinal barrier markers can be used to study the role of the gut–liver axis. The aim of this study was to gain insight into the pathogenesis of MASLD and examine the impact of bacterial translocation markers and intestinal and hepatic fatty acid-binding proteins (I-FABP and L-FABP) in children with MASLD. **Method**: We examined 60 children with MASLD and overweight/obesity (MASLD was diagnosed based on increased liver echogenicity in ultrasound and elevated ALT activity), aged 14.5 years (range 8.5 to 15.8); 33 children with overweight/obesity without MASLD, aged 13.0 years (range 11.4 to 15.8); and 16 healthy controls aged 11.0 years (range 7.0 to 16.2). Defensin, I-FABP, and L-FABP levels were measured using commercial kits: ELISA kits (Drg Medtek) were used to assess α-5 and α-6 defensin concentrations (HBD5, HBD6). I-FABP and L-FABP concentrations were measured using commercial ELISA kits (Hycult Biotech Inc., Wayne, PA, USA). ANOVA analysis was used to compare results across the three study groups. **Results**: A significant difference was found for the following tests among children with MASLD, obesity, and healthy controls: defensin 6 (14.4 ng/mL vs. 6.13 ng/mL vs. 17.2 ng/mL, respectively), L-FABP (9168 pg/mL vs. 7954 pg/mL vs. 7620 pg/mL, respectively), and I-FABP (272 pg/mL vs. 321 pg/mL vs. 330 pg/mL, respectively). No differences were found in defensin 5 levels (median 567.2 pg/mL vs. 485.7 pg/mL vs. 601.8 pg/mL). No differences were observed in cholesterol levels (HDL, LDL) or triglyceride concentrations, as well as apolipoprotein levels. **Conclusions**: Based on our study, it was concluded that inflammation and intestinal barrier damage lead to increased L-FABP levels, as it is released from enterocytes in response to oxidative stress or tissue damage. Defensin 6 may indirectly affect L-FABP through microbiota regulation and protection of the intestinal barrier. Defensin 6 also exerts antimicrobial activity and may accompany liver inflammation, with its increased concentration in comparison to obesity explained by the activation of defense mechanisms.

## 1. Introduction

**Figure 1:**
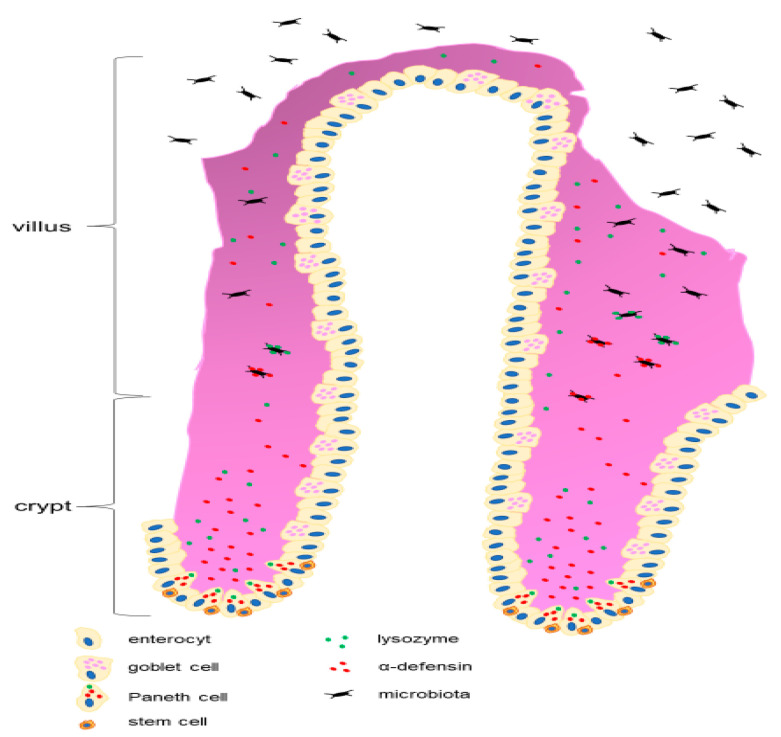
In the small intestine: α-defensins, microbiota, stem cells, and Paneth cells.

**Figure 2:**
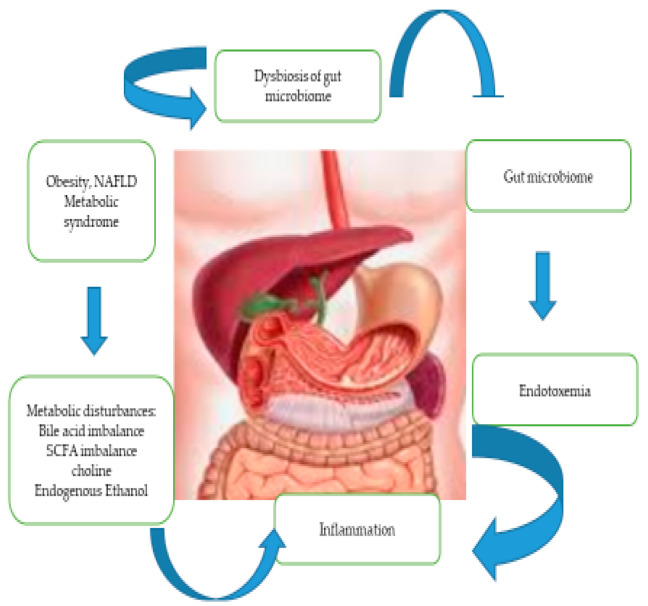
Interactions between defensins, gut microbiota, and liver pathophysiology in patient with MASLD.

The definition of metabolic dysfunction-associated steatotic liver disease (MASLD) indicates insulin resistance (IR), dyslipidemia, and dysbiosis. Dysbiosis can be caused by overeating, especially fructose, leading to impaired gut function in the form of intestinal inflammation, changes in microbiota, and alterations in cell function, particularly Paneth cells of the small intestine (Figure 1). The role of Paneth cells is to regulate microbial diversity by expressing antimicrobial peptides, specifically human α-defensin-5 (DEFA5). Intestinal defensins, known as DEFA5 and defensin 6 (DEFA6), or alpha-defensins 5 and 6, are types of antimicrobial peptides that occur in myeloid and Paneth cells. Rather than directly killing the cells, DEFA6 seems to capture bacteria. Intestinal epithelial cells, by absorbing nutrients essential for life, also prevent pathogen invasion [1,2]. The mechanisms of transcellular bacterial translocation are the result of endocytosis and transcytosis. This mechanism allows bacteria to be engulfed by enterocytes via endocytosis and then transported in vesicles to the opposite side of the cell (transcytosis). Some bacteria, such as Shigella and Listeria monocytogenes, secrete proteins that modify the actin of enterocytes, enabling them to enter the cell and further migrate intracellularly, causing cytoskeletal rearrangement. Some bacteria can survive in enterocytes by avoiding destruction by lysosomes. Clostridium difficile bacteria can damage enterocytes through toxins, leading to cell death and facilitating translocation and induction of enterocyte apoptosis [3,4]. The intestinal epithelium is the largest body surface chronically exposed to various pathogens, toxins, and commensal microbiota. The lumen of the human intestine is home to over 1×10¹⁴ bacteria, and the normal intestinal microbiota consists of a vast number of symbiotic microorganisms [5]. Children are an interesting research group because tests to assess the gut flora can be performed in high-risk children, such as those with obesity, insulin resistance or a family history of MASLD. Markers like defensins 5/6, I-FABP, and L-FABP were selected because they represent the front lines of the gut–liver connection: defensins to mucosal defense and microbiota balance. I-FABP regulates gut epithelial injury; L-FABP participates in liver inflammation and fat handling. Together, they help detect early, subclinical changes in the gut–liver axis that may contribute to MASLD and other childhood metabolic disorders. These insights are particularly valuable for early diagnosis, risk stratification, and targeted intervention in pediatric populations. Defensins 5 and 6, I-FABP and L-FABP in children were chosen to assess the translocation of bacterial flora because they provide specific information on intestinal integrity, microbial defense and liver–gut interactions, which are very important in the development and progression of metabolic disorders in children, especially metabolic dysfunction-associated steatotic liver disease (MASLD) [6,7]. Fluctuating levels of defensins may indicate impaired mucosal immunity, increasing intestinal permeability (“leaky gut”) and allowing microbial products such as lipopolysaccharides (LPS) to enter the bloodstream—causing inflammation and contributing to liver pathology. I-FABP (intestinal fatty acid-binding protein) is released into the bloodstream when enterocytes (cells of the intestinal lining) become damaged, and is a sensitive biomarker of intestinal epithelial damage. L-FABP (liver fatty acid-binding protein) reflects hepatic lipid metabolism and may also increase in response to oxidative stress or liver inflammation. In pediatric MASLD, elevated L-FABP levels may indicate early liver involvement and metabolic stress. They regulate the immune system in the gut by affecting the activity of innate immune cells, such as macrophages and dendritic cells. By modulating the immune response locally, defensins can help prevent excessive inflammation that could disrupt the intestinal barrier. Defensins 5 and 6 show promise as early biomarkers and therapeutic targets for pediatric MASLD. Children are an interesting research group because tests to assess the gut flora can be performed in high-risk children, such as those with obesity, insulin resistance or a family history of MASLD. I-FABP is released into the bloodstream when enterocytes (cells of the intestinal lining) become damaged, and is a sensitive biomarker of intestinal epithelial damage. Elevated levels of I-FABP in children may signal intestinal barrier dysfunction, a key feature of the imbalance of the gut–liver axis in MASLD in show Figure 2. L-FABP reflects hepatic lipid metabolism and may also increase in response to oxidative stress or liver inflammation. In pediatric MASLD, elevated L-FABP levels may indicate early liver involvement and metabolic stress [8,9]. Altered microbiota affects short-chain fatty acid production, bile acid metabolism, and immune signaling. LPS and other microbial products activate Kupffer cells (liver macrophages), triggering chronic low-grade inflammation. Inflammatory cytokines and gut-derived metabolites interfere with insulin signaling, which is a key player in metabolic syndrome and hepatic steatosis. L-FABP binds long-chain fatty acids and participates in their transport to mitochondria or cell nuclei. An increase in free fatty acids is associated with impaired insulin action (lipotoxicity). Oxidative stress and inflammation lead to disruption in L-FABP expression, which can lead to lipid accumulation and increased oxidative stress in hepatocytes, increasing the risk of insulin resistance and MASLD. L-FABP appears in the urine with renal tubular damage, which often accompanies metabolic syndrome.

The epithelial cells lining the small intestine are organized into villi and crypts, consisting of four main, terminally differentiated cell types: columnar cells, enteroendocrine cells, goblet cells, and Paneth cells (Figure 1). These cell lines are generated by cells called crypt base columnar (CBC) cells, located at the bottom of the crypt, next to Paneth cells [6]. Intestinal epithelial cells, except for Paneth cells, migrate toward the upper part of the villi as they differentiate and renew themselves every three to four days [10]. Paneth cells, however, are located at the base of the crypts in the small intestine, making physical contact with CBC stem cells. Fatty acid-binding proteins (FABP) are a group of cytoplasmic proteins with a small molecular mass involved in binding long-chain free fatty acids and directing them for intracellular use or storage in the cytoplasmic reticulum [11]. The name of these proteins comes from the site where they were first identified. L-FABP (liver fatty acid-binding protein) was first detected in hepatocytes, but this protein is also present in small and large intestinal cells, lungs, and kidneys. I-FABP (intestinal fatty acid-binding protein) is produced by mature enterocytes and is known as a marker of cell necrosis. The concentrations of L-FABP and I-FABP change in the blood due to liver or intestinal damage caused by intestinal microbiota translocation [9]. Fatty acid-binding proteins (FABP) can be secreted extracellularly in a soluble form, but they are commonly studied as plasma markers of cell rupture or death. FABPs are released into the blood and/or urine after tissue damage, and they can be measured within a few hours using an enzyme-linked immunosorbent assay (ELISA). We hypothesized that sampling for the measurement of L-FABP and I-FABP levels, as well as defensins 5 and 6 in the serum of children with obesity and MASLD, might explain the cause of metabolic disturbances in children with obesity and MASLD and confirm or disprove whether intestinal microbiota translocation could influence the development of obesity and MASLD. As is known, inappropriate nutrition, including a high-fat diet (HF), can induce intestinal dysbiosis and alter host homeostasis, leading to mucosal inflammation and metabolic endotoxemia [10]. The HF diet increases circulating bile acids [11] and affects the gut bacterial community by altering the bile acid profile [12]. After a meal, cholic acid (CA) and chenodeoxycholic acid are secreted into the duodenum, where they facilitate fat absorption. In the distal ileum and colon, deconjugation and dehydroxylation occur due to certain gut bacteria, creating secondary bile acids, such as deoxycholic acid (DCA) and lithocholic acid (LCA) [13]. Bile acids, especially DCA, have strong antimicrobial and cytotoxic effects, thus regulating the microbiota composition and host physiology. Bile acids exert their action through the activation of nuclear and plasma membrane receptors [14]. The G protein-coupled receptor TGR5 is a plasma membrane receptor for bile acids, widely distributed in the gastrointestinal tract [15]. TGR5 regulates bile acid synthesis, intestinal secretion, glucose homeostasis, energy expenditure, gastrointestinal motility, and inflammation. TGR5 receptors are present on Paneth cells, which are highly specialized epithelial cells located at the base of the Lieberkühn crypts of the small intestine. Paneth cells secrete antimicrobial peptides into the crypt lumen, protecting the host from intestinal pathogens, helping shape the composition of colonizing bacteria, protecting against bacterial translocation across the epithelium, and acting as immune modulators and trophic factors [16]. Paneth cell dysfunction has been linked to changes in the gut bacterial composition in obese individuals [17,18].

Defensins are particularly crucial in maintaining the balance of microbial populations in the gut, preventing pathogen overgrowth, and modulating inflammation. The gut–liver axis is an intricate communication system where signals between the gut microbiota and the liver contribute to metabolic and immune homeostasis. Dysregulation of this axis has been linked to various metabolic disorders of childhood, such as MASLD and obesity, and the role of defensins is an emerging area of research. In children with MASLD and obesity, the gut microbiota often exhibits an imbalance (dysbiosis) that can affect both overall gut immunity and systemic inflammation. Given the antimicrobial properties of defensins, alterations in their levels or activity could potentially reflect disturbances in the gut microbiota composition or function. This could lead to a compromised intestinal barrier, allowing endotoxins and inflammatory mediators (such as lipopolysaccharides) to enter circulation and affect the liver, thereby contributing to metabolic dysfunction and the development of liver diseases [19,20]. Defensins regulate microbial populations in the gut. Dysbiosis in children with obesity and MASLD could affect defensin production or function, which in turn may contribute to gut inflammation and liver injury. Defensins help maintain the integrity of the intestinal epithelium. In conditions like MASLD and obesity, a weakened barrier (due to reduced defensin levels or altered function) might allow for the translocation of harmful bacteria or their metabolites (like endotoxins) into the bloodstream, leading to systemic inflammation and liver damage. Defensins are involved in the regulation of inflammation [21]. In obesity and MASLD, chronic low-grade inflammation is a key feature, and defensins could either contribute to or mitigate this process, depending on their levels and activity. If defensins are disrupted, this could exacerbate inflammation, not just in the gut but also in the liver, perpetuating the metabolic dysfunction. The gut and liver communicate via various pathways, including the portal vein. Defensins might influence this communication, particularly in the setting of dysbiosis, by affecting the microbial signals that are sent from the gut to the liver. An imbalance in defensins could lead to a disrupted gut–liver axis, potentially exacerbating conditions like MASLD and obesity.

## 2. Materials and Methods

### 2.1. Patients and Study Design

The study group consisted of pediatric patients with obesity, MASLD and a control group under the care of a tertiary referral center (Department of Gastroenterology, Hepatology, Nutritional Disorders and Pediatrics, The Children’s Memorial Health Institute (IPCZD, Warsaw, Poland) who participated in follow-up visits between January 2017 and February 2018. Diagnosis was based on increased BMI, ALT transaminase activity and ultrasound findings. The following inclusion criteria were used: age (8–16 years), asymptomatic or mild/moderate liver disease, normal or slightly elevated aminotransferases). The exclusion criterion was the lack of consent to participate in the study. We conducted a cross-sectional study in 32 patients with obesity (16 girls and 16 boys) and 32 with MASLD (10 girls and 22 boys), from January 2017 to February 2018. The control group consisted of 33 healthy children with a mean age of 13.2 years (7 girls, age; 26 boys, age range (7.0–16.2 years) who were included in a pilot study to assess defensin 5 and 6 levels.

In this exploratory study we aimed to include approximately 30 patients with obesity, 30 patients with MASLD and 30 healthy controls.

The upper limit of normal ALT values for healthy children was set at 25.8 U/L (boys) and 22.1 U/L (girls). The mean age of the study participants was 13.2 years (ranging from 8.5 to 15.8) for children with MASLD, 13.3 years (11.4–15.8) for the obesity group, and 13.3 years (7.0–16.2) in the control group. The patients included in the study were of similar age, with an average age of approximately 13.3 years. The study was conducted in accordance with the Declaration of Helsinki and with the approval of the Ethics Committee of the Memorial Health Institute for Child Health. All patients and parents provided informed consent for participation in the study. The exclusion criteria were as follows: viral and autoimmune liver damage, storage disease, liver cirrhosis, type 1 and type 2 diabetes, chronic gastrointestinal diseases, and the use of lipid-lowering drugs or medications affecting lipid metabolism.

### 2.2. Blood Processing

Patients and healthy adolescents underwent a general examination, including anthropometry with BMI calculation. Venous blood was collected from the antecubital vein after at least 12 h of fasting. Blood samples were collected as early as possible into vacuum tubes; after centrifugation, part of the serum was used for immediate testing, while the remaining serum was stored at −80 °C for the determination of FABP and defensins. This blood was used to measure total cholesterol (TC), triglycerides (TG), low-density lipoprotein cholesterol (LDL-C), high-density lipoprotein cholesterol (HDL-C), gamma-glutamyl transferase (GGT), alanine aminotransferase (ALT), aspartate aminotransferase (AST), and high-sensitivity C-reactive protein (hs-CRP) using enzymatic commercial biochemical tests (Roche Diagnostics; Mannheim Germany). Glucose levels were measured using the hexokinase method, while insulin levels were determined using chemiluminescence assays (Siemens Healthcare Diagnostics, Swords Co Dublin, Ireland). Insulin resistance was calculated using the following formula: (fasting glucose × fasting insulin)/22.5, in accordance with the homeostatic model assessment of insulin resistance (HOMA-IR).

#### Determination of Defensin 5, 6, L-FABP and I-FABP

The levels of I-FABP, L-FABP and defensin 5 and 6 were measured using commercially available enzyme-linked immunosorbent assays: Human L-FABP and I-FABP ELISA Kit (Hycult Biotech, Uden, The Netherlands, catalog number: HK404-02 and HK406) and Human defensin 5 and 6) (DrgMedtek, catalog number: CSB-EL006657HU). The ranges of measurable L-FABP concentrations were 2479–21,000 pg/mL, for I-FABP from 10 to 1250 pg/mL, defensin 5 from 25 to 800 pg/mL and defensin 6 from 0.4 to 32 pg/mL, respectively. The kit included two types of antibodies linked to a plate with antibodies against human I-FABP or L-FABP, depending on the dedicated kit, as well as biotin-conjugated antibodies against I-FABP or L-FABP. ANOVA analysis was used to compare results among the three study groups. Optical density was measured using a microplate reader (BioTek Elx 800 Biokom, Janki, Poland) set at a wavelength of 450 nm. The mean absorbance for each set of duplicate standards and controls was calculated with reference to specific standard curves (defined separately for each protein) and was generated using computer software dedicated to the microplate reader.

### 2.3. Data Analysis

The serum concentrations of fatty acid-binding proteins (FABP) and defensins 5 and 6 did not follow a Gaussian distribution, which was verified using the Kolmogorov–Smirnov–Lilliefors test. The IQR—interquartile range—was another parameter reported. The medians for FABP and defensins 5 and 6 levels for each of the three groups were compared using the Kruskal–Wallis test, with Dunn’s multiple comparison test as the post hoc analysis. A *p*-value < 0.05 was considered statistically significant. Spearman correlation analysis was used to evaluate the relationship between FABP and defensins 5 and 6 levels, as well as biochemical parameters. Specificity, sensitivity, and characteristic curves were generated to analyze optimal cut-off levels. Sensitivity values were calculated, and characteristic curves were generated.

## 3. Results

In Table 1, the mean and standard deviation for selected biochemical parameters are presented. In the studied population, no statistically significant differences were found between the obesity, MASLD, and control groups for transaminases (ALT, AST), bilirubin, GGT, and CRP. Parameters assessing lipid disturbances, including total cholesterol (TC), triglycerides (TG), LDL-C, and HDL-C, also did not show statistically significant differences. Apolipoproteins AI, B, and VLDL-C differed significantly between the groups, with the highest concentrations of apo AI and VLDL-C observed in the MASLD patient group compared to the other groups. A higher concentration of apo B was observed compared to the other parameters measured. Insulin resistance, expressed as the HOMA-IR index, was observed in the groups of children with obesity and MASLD, even though the values for glucose and insulin were within the upper range of reference values. The concentration of uric acid was within the range of reference values.

The analysis of the operating characteristic curve of the studied parameters indicating the gut–liver microbiota translocation shows the effectiveness of the prediction model by plotting the qualitative characteristics of the binary classifiers obtained from the model using different cut-off points. In children with MASLD, the prediction of the presence or absence of sensitivity for I-FABP and defensin-5, for defensin-6 and for L-FABP is presented in Figure 1 and Figure 2.

The analysis of the operational characteristic curve of the tested parameters indicating the gut–liver microbiota translocation, such as for I-FABP, shows the effectiveness of the predictive model by plotting the qualitative characteristic of binary classifiers derived from the model using various cutoff points.

The median values of L-FABP for each of the four groups were as follows: the MASLD group (9168 pg/mL, IQR 4809–25,620 pg/mL), the obesity group (7954 pg/mL, IQR 809–35,660 pg/mL), the control group (7620 pg/mL, IQR 2754–20,141 pg/mL), and the mixed group, where patients with MASLD and obesity were combined (13,573 pg/mL, IQR 809–35,660 pg/mL). The median values for L-FABP are significantly higher in the mixed group, where patients with simple obesity and MASLD were combined, as these patients, in addition to the features of fatty liver, exhibited obesity, compared to all other groups (*p* < 0.0006).

The median values of I-FABP for each of the four groups were as follows: the MASLD group (274 pg/mL, IQR 88–1588 pg/mL), the obesity group (321 pg/mL, IQR 58–1394 pg/mL), the control group (330 pg/mL, IQR 10–1258 pg/mL), and the mixed group, where patients with MASLD and obesity were combined (420 pg/mL, IQR 58–1588 pg/mL). The median values of defensin-5 for each of the four groups were as follows: the MASLD group (567.15 pg/mL, IQR 15.3–798.3 pg/mL), the obesity group (485.7 pg/mL, IQR 25.6–798.3 pg/mL), the control group (601.8 pg/mL, IQR 344.8–814.3 pg/mL), and the mixed group, where patients with MASLD and obesity were combined (643.8 pg/mL, IQR 485.7–798.3 pg/mL). The median values for I-FABP are significantly higher in the mixed group of patients with simple obesity and MASLD, compared to all other groups (*p* < 0.028).

**Figure 3:**
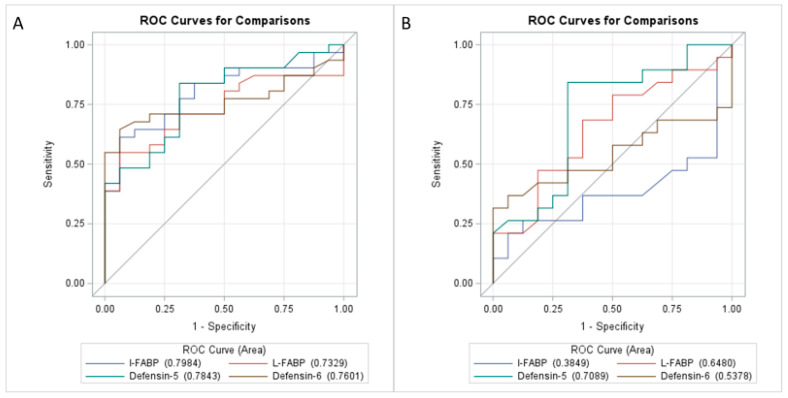
Receiver operating characteristic (ROC) curves for prediction (**A**) obesity and (**B**) MASLD from I-FABP, L-FABP, defensin-5 and defensin-6.

Figure 3 shows the increasing levels of defensin-6 in patients from the control group compared to patients with MASLD and obesity, with the lowest defensin-6 concentration observed in children with obesity. When comparing defensin-6 and L-FABP in pairs, we identified a significant difference between the obesity group and the control group (*p* = 0.005), but the difference between MASLD and the control group was not significant (*p* = 0.68). The levels of liver fatty acid-binding protein (L-FABP) in serum were significantly elevated in patients with MASLD (Figure 3) compared to the obesity and control groups.

### Figures, Tables and Schemes

**Figure 4:**
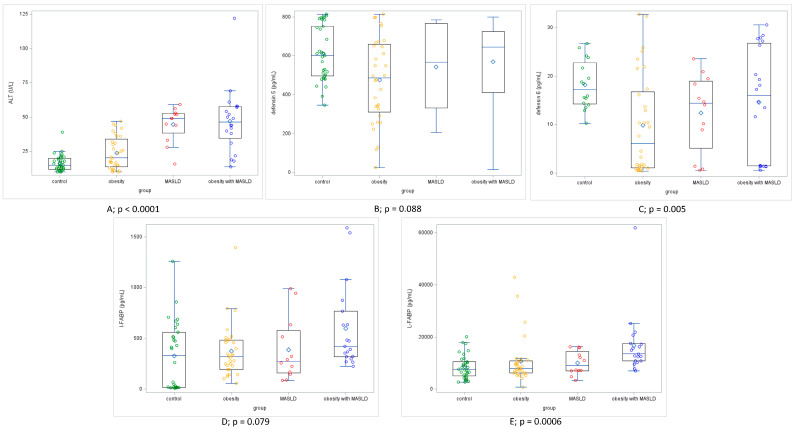
Results of (**A**) the activity of alanine aminotransferase (ALT), and the levels of biomarkers of bacterial translocation (**B**) defensin 5, (**C**) defensin 6, (**D**) I-FABP, (**E**) L-FABP in the studied groups. *p* represents *p*-values of Kruskal–Wallis test.

Figure 4 shows receiver operating characteristic (ROC) curves for the prediction of obesity and MASLD from I-FABP, L-FABP, defensin-5 and defensin-6 levels.

## 4. Discussion

These results support the hypothesis of loss of intestinal barrier integrity in MASLD, resulting in chronic low-grade inflammation and increased diffusion of bacterial components into the bloodstream. Our study demonstrated intestinal barrier dysfunction, assessed by measuring markers of intestinal damage and bacterial translocation based on the evaluation of defensins 5 and 6, as well as levels of I-FABP and L-FABP. Elevated levels of defensins can be explained by changes in the microbiota. Defensin-5 levels may affect the growth of Bifidobacterium, which was not investigated in our study, but has been documented in other studies [22]. Of these, Bifidobacterium was the most influential regulated genus, inversely correlated with hepatic IR, AUC, hepatic TG and fasting insulin levels [23]. Although beyond the scope of this report, further studies are needed to investigate the direct role of HD-5- and HD-6-induced changes in the gut microbiome and determine whether such changes improve metabolism, or whether these features can be achieved independently of the conventional innate microbiota.

We found no lipid or carbohydrate abnormalities in the study groups, but of note is the increase in VLDL-C lipoprotein and HOMA-IR index; this provides evidence for the concept of using human defensins to regulate metabolism and alleviate dyslipidemia, IR and MASLD. However, further studies are needed to elucidate the main mechanisms by which defensin-5 exerts its comprehensive functions on metabolism. Patients with MASLD are a heterogeneous group, as they have dyslipidemia, obesity, insulin resistance and other metabolic disorders in addition to hepatic steatosis. Recently, it has been suggested that changes in the gut microbiome may be responsible for these disorders. Alterations in the translocation of the intestinal microbiota appear to be associated with L-FABP and I-FABP, which are recognized early serological markers of intestinal epithelial damage in MASLD [24].

Defensin 6 may influence L-FABP indirectly through microbiota regulation and protection of the intestinal barrier if DEFA6 acts effectively, it may limit epithelial damage, thereby reducing the release of L-FABP. To our knowledge, this is the first study describing such a relationship in a pediatric cohort assessing defensins and fatty acid-binding proteins. The current study shows that I-FABP levels are significantly elevated in the group of children with MASLD. Elevated levels of defensins 5, 6, and fatty acid-binding proteins (I-FABP and L-FABP) in both MASLD and obesity patients compared to healthy controls indicate intestinal epithelial damage in both patient cohorts. However, no differences were observed between the research groups, suggesting that intestinal leakage, as measured by I-FABP levels, may be an independent predictor of MASLD development. The pediatric MASLD cohort represents early liver damage, as opposed to adult MASLD, which is why these data provide additional value compared to studies in adults. We also identified increased ferritin concentrations in the MASLD and obesity groups. Numerous publications have already indicated ferritin as a longitudinal risk factor for MASLD and its progression. Therefore, ferritin in serum could potentially serve as a less invasive and more effective biomarker for predicting disease progression. In studies of Escherichia coli exposed to DEFA5, morphological changes occurred, including cell elongation and clumping, occupying the first line of the human interface with the external environment, which is constantly stimulated by food and microorganisms, and therefore participates in nutrient absorption processes, regeneration, and mucosal immunity [24]. α-defensins 5 and 6 can kill or inhibit the growth of a wide range of pathogens, including bacteria, fungi, and viruses. Gut barrier integrity refers to the physical and functional barriers that prevent harmful substances, pathogens, and toxins from entering the bloodstream. The intestinal epithelium, including tight junctions and mucosal secretions, forms this barrier. α-defensins 5 and 6 regulate the immune system in the gut by influencing the activity of innate immune cells such as macrophages and dendritic cells. By modulating the local immune response, defensins may help prevent excessive inflammation that can disrupt the gut barrier. Immune modulation is where α-defensins 5 and 6, through their interaction with dendritic cells and other immune cells in the gut, can help promote tolerance to commensal bacteria while also activating immune responses when needed to fight infection. This helps maintain a homeostatic immune environment, which is critical for preventing both infections and autoimmune disease. Interaction with tight junctions and mucosal immunity is where defensins can affect the expression of tight junction proteins in the intestinal epithelium. Tight junctions are proteins that seal the spaces between epithelial cells, preventing harmful substances from leaking into the bloodstream. Modulation of inflammation and prevention of dysbiosis through α-defensins 5 and 6 can modulate local inflammation in the gut [25]. If these defensins are deficient or not functioning properly, this can result in increased microbial dysbiosis and chronic inflammation, which contribute to disorders such as obesity and metabolic syndrome. In the context of a dysbiotic microbiota, the lack of sufficient antimicrobial peptides such as α-defensins can lead to the overgrowth of pathogenic microbes and intestinal permeability, commonly referred to as “leaky gut.” This can promote the translocation of bacteria and their byproducts into the systemic circulation, potentially causing systemic inflammation and liver damage (as seen in metabolic disorders) [26].

Defensin levels (particularly α-defensins 5 and 6) may be closely linked to changes in the composition of the intestinal microbiota. Dysbiosis, an imbalance in the gut microbiota, has been linked to the development of these metabolic disorders. Defensins help maintain the balance of the microbiota, and changes in their levels may reflect or contribute to the specific microbiota changes that exacerbate MASLD and obesity. Changes in the specific microbiota may be associated with altered defensin levels in MASLD and obesity in an increased Firmicutes/Bacteroidetes ratio, but Firmicutes and Bacteroidetes are the two most dominant groups in the gut. An elevated Firmicutes to Bacteroidetes ratio has been commonly observed in individuals with obesity. Decreased α-defensins, such as in states of gut dysbiosis, may allow for the overgrowth of Firmicutes (which are associated with higher energy extraction from the diet) at the expense of Bacteroidetes. This shift in microbial composition could contribute to increased caloric absorption, promoting fat accumulation and obesity. Defensins help regulate this balance by controlling bacterial populations, and a decrease in defensin expression could reduce the gut’s ability to keep pathogenic or energy-harvesting bacteria in check. Defensins play a key role in controlling the growth of pathogenic bacteria (e.g., Enterobacteriaceae, Clostridium difficile). A reduction in defensin levels may lead to an increase in these harmful microbes [27]. In MASLD and obesity, an overgrowth of pathogenic microbes can contribute to intestinal inflammation and intestinal permeability (leaky gut), allowing bacterial products like lipopolysaccharides (LPS) to enter circulation, which can trigger systemic inflammation and contribute to liver dysfunction. Imbalance in Short-Chain Fatty Acid (SCFA)-producing Bacteria, such as butyrate, acetate, and propionate, are produced by Bacteroidetes and Firmicutes through the fermentation of dietary fiber. SCFAs have anti-inflammatory properties and help maintain gut barrier integrity. Defensin levels influence the microbial composition that produces these beneficial metabolites. A deficiency in defensins may alter the populations of SCFA-producing bacteria, leading to a decrease in SCFA production. This may impair gut barrier function, promote inflammation, and contribute to liver fat accumulation in MASLD. Butyrate-producing bacteria, in particular, are beneficial for gut health and have been shown to regulate intestinal permeability and immune responses. Reduced defensins might lead to a reduction in these beneficial microbes, exacerbating the gut–liver axis dysfunction. Lipopolysaccharides (LPS), produced by Gram-negative bacteria like Escherichia coli and other members of the Enterobacteriaceae family, can translocate from the gut into the bloodstream when the intestinal barrier is compromised [28]. In MASLD and obesity, a weakened gut barrier (due to lower defensin levels) may allow for LPS translocation. The presence of LPS in circulation can trigger systemic inflammation, liver damage, and insulin resistance, all of which contribute to the progression of obesity and MASLD. A decrease in α-defensins 5 and 6 could promote the growth of LPS-producing bacteria, which may further exacerbate metabolic dysfunction and liver disease. Some gut microbes are specifically involved in regulating immune responses, promoting immune tolerance to commensal bacteria and limiting excessive inflammation. Lactobacillus and Bifidobacterium species are examples of beneficial microbes involved in immune modulation. A reduction in defensins could lead to a decline in the abundance of these immune-modulating bacteria. This may lead to an imbalance in the gut-associated immune system, contributing to chronic low-grade inflammation, which is a hallmark of obesity and MASLD. Microbial diversity is generally considered a marker of a healthy gut. In individuals with MASLD and obesity, lower microbial diversity has been observed, which is often associated with dysbiosis and increased susceptibility to metabolic disease [29].

Changes in defensin levels could serve as indicators of gut barrier dysfunction and microbiota imbalances. For example, low defensin levels might indicate a weakened intestinal barrier, which could lead to increased microbial translocation, promoting systemic inflammation and contributing to liver damage in MASLD. Elevated defensin levels, on the other hand, might indicate overactive immune responses in the gut. Defensin levels could thus be used as early biomarkers to detect dysbiosis or intestinal permeability, both of which are linked to the progression of metabolic diseases. Monitoring defensin levels could help track disease progression in individuals with MASLD or obesity, as changes in defensin expression may correlate with shifts in gut microbiota and inflammation status. Similarly, therapeutic interventions that aim to modulate defensin levels (either by boosting or inhibiting their activity) could be tracked using defensin levels as biomarkers to assess treatment efficacy.

Since defensins play a role in maintaining the integrity of the gut–liver axis; enhancing defensin activity could potentially protect the liver from the effects of microbial translocation and the resulting systemic inflammation. This could be particularly beneficial in treating MASLD; therapeutics could be developed to target microbial dysbiosis in the gut, thereby modulating the gut’s immune environment and reducing liver fat accumulation and inflammation [30].

Therapeutic strategies to modulate defensins in the treatment of MASLD can be based on dietary and lifestyle interventions. Dietary modification to increase defensins should include foods rich in fiber and polyphenols (e.g., fruits, vegetables, whole grains), as they improve Paneth cell function and defensin production. Prebiotics and probiotics (e.g., Lactobacillus, Bifidobacterium) can rebalance the intestinal microbiota and stimulate the expression of defensins.

Pharmacological approaches can be based on defensin-inducing therapies (e.g., small-molecule Paneth cell activators) which can help restore the integrity of the gut–hepatic axis. Combination therapy with existing MASLD drugs (e.g., GLP-1 receptor agonists) can be used to achieve synergistic effects.

The standardization of defensin testing methods (fecal vs. serum); need for large pediatric studies to establish diagnostic thresholds; and the cost-effectiveness and accessibility of defensin-based tests are of note here. Implementation strategies include pilot studies in pediatric MASLD clinics to assess feasibility, the incorporation of defensin biomarkers into MASLD guidelines for early detection, and the development of rapid, cost-effective defensin assays for clinical use.

## 5. Conclusions

In conclusion, we can speculate that there is no significant intestinal cell damage in MASLD, as reflected by the I-FABP levels, which were similar across all groups. The variable levels of defensin 5 may reflect changes in the gut microbiome and metabolome, which are different in obese children with MASLD. It plays a protective role by neutralizing pathogens and modulating the gut microbiota. DEFA6 may influence L-FABP indirectly through microbiota regulation and intestinal barrier protection, and if DEFA6 acts effectively, it may limit epithelial damage, thus reducing the release of L-FABP. Therefore, DEFA6 plays a crucial role in maintaining intestinal barrier integrity and regulating microbiota, which may indirectly influence L-FABP levels. Since defensin 6 exerts antimicrobial activity and may accompany liver inflammation, its increased concentration compared to obesity can be explained by the activation of defensive mechanisms. Our preliminary results suggest a potential role for defensins in describing and studying the gut–liver axis and intestinal barrier in patients with obesity and MASLD. Evidence from this study suggests that I-FABP, L-FABP, and defensins 5 and 6 may not serve as potential early biomarkers for MASLD diagnosis, but they can be used to describe organ damage such as liver and intestinal damage in children with MASLD. In MASLD and obesity, changes in defensin levels (such as α-defensins 5 and 6) may lead to an altered gut microbiota composition that promotes dysbiosis, systemic inflammation, and disruption of gut barrier integrity. This dysbiosis, in turn, can exacerbate the progression of metabolic diseases, including MASLD, by fostering an environment that enhances pathogen overgrowth, increased intestinal permeability, and impaired immune regulation. Understanding these connections could provide insight into potential therapeutic strategies for these conditions, including the modulation of defensin levels or microbiota composition.

## 6. Limitations and Strengths

We are aware of some limitations of our study. First, it was limited by the relatively small patient group in a single center. Second, we did not perform a sample size estimation, which was not possible based on the available data. Therefore, negative results should be interpreted with caution. MASLD in children is rarely an indication for liver biopsy, so we did not obtain data on liver damage, except for liver laboratory parameters. We did not investigate the gut microbiota, which is a complex issue that is not easily correlated with biochemical parameters. However, the study has significant strengths as it is the first pediatric study analyzing defensins and FABP in MASLD. Additionally, we believe it is worth noting that in our study epithelial damage was investigated using four independent markers (DEFA5, DEFA6, I-FABP, L-FABP).

## Figures and Tables

**Table 1:** Mean levels of biochemical parameters in the MASLD group in relation to children with obesity and the control group.

Variable	Obesity Group	MASLD	Control Group	*p*-Value
ALT [U/L]	28.4 ± 20.8	34.9 ± 19.2	18.6 ± 8.3	NS
AST [U/L]	26.0 ± 11.2	26.6 ± 10.4	23.4 ± 3.2	NS
GGTP [U/L]	21.0 ± 10.4	25.9 ± 12.5	23.4 ± 0.2	NS
bilirubin [mg/dL]	0.65 ± 0.2	0.67 ± 0.4	0.66 ± 0.01	NS
CRP [mg/dL]	0.60 ± 0.2	0.62 ± 0.2	0.56 ± 0.2	NS
TCH [mg/dL]	167.1 ± 34.2	168.9 ± 39.6	161.5 ± 6.3	NS
TG [mg/dL]	99.9 ± 55	99.4 ± 43.3	90.8 ± 5.4	NS
LDL-C [mg/dL]	96 ± 26.7	96.2 ± 28.8	94.1 ± 12.9	NS
VLDL-C [mg/dL]	19.9 ± 11.1	20.1 ± 8.7	17.4 ± 1.2	<0.05
HDL-C [mg/dL]	51.1 ± 12.8	53.3 ± 16	50.50 ± 5.7	NS
apolipoprotein AI [g/L]	1.38 ± 0.3	1.41 ± 0.4	1.26 ± 0.2	<0.05
apolipoprotein B [g/L]	0.98 ± 1.9	0.74 ± 1.3	0.71 ± 0.1	<0.05
ferritin [ng/mL]	75.3 ± 21.9	75.7 ± 20.5	57.0 ± 17.5	<0.05
glucose [mg/dL]	90.3 ± 5.3	88.2 ± 11.4	87.3 ± 1.3	NS
insulin [μmol/L]	14.9 ± 3.2	14.6 ± 4.9	11.9 ± 0.9	<0.05
HOMA IR	3.30 ± 0.7	3.2 ± 1.1	2.62 ± 0.2	<0.05
UA [mg/dL]	4.79 ± 1.0	4.57 ± 1.1	4.3 ± 0.8	NS

Abbreviations: ALT, Alanine Aminotransferase; AST, Aspartate Aminotransferase; GGT, Gamma-Glutamyltransferase; HOMA-IR, Homeostasis Model Assessment of Insulin Resistance; CRP, C-Reactive Protein; serum ferritin; UA, Uric Acid; TCH, total cholesterol; TG, triglicerides; HDL-C, high-density lipoprotein cholesterol; LDL-C, low-density lipoprotein cholesterol.

## Data Availability

The data are available upon request.
